# Blast Overpressure-Induced Neuroinflammation and Axonal Injury in the Spinal Cord of Ferrets

**DOI:** 10.3390/brainsci15101050

**Published:** 2025-09-26

**Authors:** Gaurav Phuyal, Chetan Y. Pundkar, Manoj Y. Govindarajulu, Rex Jeya Rajkumar Samdavid Thanapaul, Aymen Al-Lami, Ashwathi Menon, Joseph B. Long, Peethambaran Arun

**Affiliations:** 1 Blast-Induced Neurotrauma Branch, Center for Military Psychiatry and Neuroscience, Walter Reed Army Institute of Research, Silver Spring, MD 20910, USA; gaurav.phuyal.ctr@health.mil (G.P.); chetan.y.pundkar.ctr@health.mil (C.Y.P.); manoj.y.govindarajulu.ctr@health.mil (M.Y.G.); rexjeyarajkumar.samdavidthanapaul.ctr@health.mil (R.J.R.S.T.); aymen.k.al-lami.ctr@health.mil (A.A.-L.); ashwathimenon05@gmail.com (A.M.); joseph.b.long.civ@health.mil (J.B.L.); 2 College of Natural and Mathematical Sciences, University of Maryland, Baltimore County, Baltimore, MD 21250, USA

**Keywords:** blast exposure, ferret, cervical spinal cord, gait performance, neuroinflammation, axonal injury

## Abstract

Background: Blast-induced spinal cord injuries (bSCI) account for 75% of all combat-related spinal trauma and are associated with long-term functional impairments. However, limited studies have evaluated the neuropathological outcomes in the spinal cord following blast exposure. Objectives In this study, we aimed to determine the acute and sub-acute neuropathological changes in the spinal cord of ferrets after blast exposure. Methods: An advanced blast simulator was used to expose ferrets to tightly coupled repeated blasts. The Catwalk XT system was used to detect gait performances in ferrets at 24 h and 1 month post-blast exposure. After euthanasia, the cervical spinal cord samples were collected at 24 h or 1 month post-blast. A quantitative real-time polymerase chain reaction was performed to evaluate changes in the gene expression of multiple Toll-like Receptors (*TLR*), Cyclooxygenase (*COX-1* and *COX-2*) enzymes and cytokines. Western blotting was performed to investigate markers of axonal injury (Phosphorylated-Tau, pTau; Phosphorylated Neurofilament Heavy Chain, pNFH; and Neurofilament Light Chain present in degenerating neurons, NFL-degen) and neuroinflammation (Glial Fibrillary Acidic Protein, GFAP; and Ionized Calcium Binding Adaptor Molecule, Iba-1). Results: Blast exposure significantly affected the gait performances in ferrets, especially at 24 h post-blast. Multiple TLRs, COX-2, Interleukin-1-beta (*IL-1β*), Interleukin-6 (*IL-6*), and Tumor Necrosis Factor-α (TNF-α) were significantly upregulated in the spinal cord at 24 h after blast exposure. Although only *TLR3* was significantly upregulated at 1 month, non-significant increases in *TLR1* and *TLR2* were observed in the spinal cord at 1 month post-blast. Phosphorylation of Tau at serine (Ser396 and Ser404) and threonine (Thr205) increased in the spinal cord at 24 h and 1 month post-blast exposure. The increased expression of pNFH and NFL-degen proteins was evident at both time points. The expression of GFAP, but not Iba-1, significantly increased at 24 h and 1 month following blast exposure. Conclusions: Our results indicate that blast exposure causes acute and sub-acute neuroinflammation and associated axonal injury in the cervical spinal cord. These data further suggest that inhibition of *TLR*s and/or *COX-2* enzyme might offer protection against blast-induced injuries to the spinal cord.

## 1. Introduction

Blast exposure is responsible for approximately 75% of combat-related spinal cord injuries, contributing significantly to morbidity and mortality rates [1,2,3]. The increasing use of improvised explosive devices (IEDs) in modern military conflicts has heightened the necessity of focusing research on blast-induced spinal cord injuries (bSCI) [4]. Such blast events often cause permanent damage to spinal cord tissue, resulting in dysfunctions of both sensorimotor and autonomic nervous systems [5]. The pathological changes following bSCI can occur immediately after the blast and may persist for many months, leading to long-term functional impairments. While a substantial body of research has investigated the effects of blast exposure on the brain, relatively few studies have addressed the pathological processes occurring within the spinal cord after blast injury.

Neuroinflammation resulting from central nervous system injury is the response by glial cells (astrocytes and microglia) as a component of the innate immune system. These activated glial cells produce pro-inflammatory cytokines, enzymes, and adhesion molecules—a response facilitated by Toll-like Receptors (*TLR*s) [6]. Functional whole-blood gene expression analyses in individuals with spinal cord injury unrelated to blast have shown an upregulation of genes involved in the pro-inflammatory *TLR* signaling pathway [7]. In a mouse model of moderate spinal contusion injury, multiple *TLR* genes (*TLR1*, *2*, *4*, *5*, and *7*) were upregulated along with associated *TLR* signaling (MyD88 and NFκB) [8]. Transcriptomic analysis in a rat model of spinal cord contusion injury revealed an increase in *TLR2* and *TLR4* expression, as well as an activation of downstream signaling pathways leading to pro-inflammatory cytokine synthesis at 2 weeks post-injury [9]. However, the association between bSCI and the neuroinflammatory phenomenon mediated by TLR signaling pathway has not been elucidated so far.

Recent findings regarding bSCI have shown alterations in axonal damage characterized by neurofilament light chain (NFL) reactive enlarged axons, vacuolar changes, and elevated levels of β-Amyloid Precursor Protein (β-APP) at 6 h and 24 h after blast injury in rats [10]. Similarly, Czeiter et al. demonstrated traumatic axonal injury markers (APP and RMO-14 immunostaining) in the thoracic spinal cord in a rat model of head impact acceleration-induced traumatic brain injury (TBI) [11]. The exposure to blast overpressure (50.3 MPa) waves concentrating on the dorsal thoracic spinal cord (T9–T10) was also demonstrated to induce necrosis and apoptosis in the lesion site in rats [12]. Following axonal injury, several neuronal proteins are released into the interstitial spaces, which then diffuse into the cerebrospinal fluid (CSF) and blood. Several studies indicated that neurofilament and Tau proteins are reliable markers of axonal injury [13,14]. However, the relationship between bSCI and axonal injury markers at acute, sub-acute as well as chronic time points need to be investigated as the limited studies on bSCI were focused only on the acute time points.

Rodents have traditionally been used to study the mechanisms of neuronal injury and to test potential therapies, but translating findings from these models to human clinical success has been challenging. One factor contributing to this gap is the significant anatomical differences between rodent and human brains. Unlike rodents, whose brains are smooth and lack folds (lissencephalic) with relatively little white matter, the human brain features complex folding (gyri and sulci) and extensive white matter regions. Additionally, the hippocampus is located differently in rodents, positioned more dorsally, compared to its temporal lobe location in humans, making it more susceptible to injury in traditional rodent TBI models. While large gyrencephalic animals such as pigs, sheep, and non-human primates better resemble human brain architecture, their use in spinal cord injury research is limited due to high costs, housing, and ethical concerns. To address some of these limitations, ferrets have gained attention as an alternative animal model given their closer genetic similarity to humans [15]. Prior work from our group has utilized ferrets to study blast-induced traumatic brain and neurosensory system injuries [16,17,18]. In this study, we expand the use of the ferret model to investigate the neuropathological mechanisms of bSCI.

## 2. Materials and Methods

Animals: Male ferrets, aged 3 to 4 months, were used. Animals were pair-housed with free access to food and water in a facility accredited by AAALAC International at the Walter Reed Army Institute of Research (WRAIR), following a standard 12 h light–dark cycle. All experiments were conducted with approved protocols from the Institutional Animal Care and Use Committee and adhered to the National Academy of Sciences’ Guide for the Care and Use of Laboratory Animals. Ferrets were randomly allocated into either sham or tightly coupled repeated blast exposure groups, with each group comprising 4–8 ferrets per test/time point.

**Figure 1:**
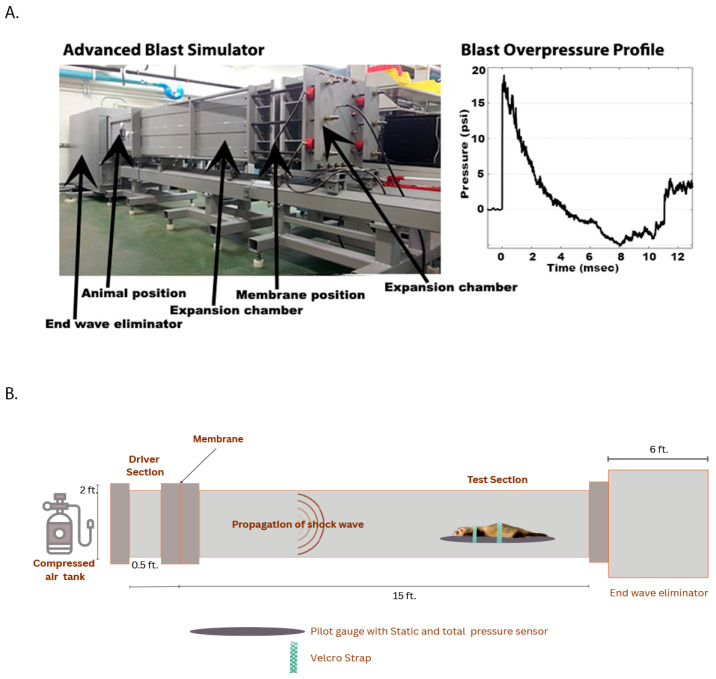
Setup and profile of blast exposure in the advanced blast simulator (ABS) at the Walter Army Institute of Research. (**A**). Photograph of the ABS facility [19], showing the designated positions for animal placements, membrane, expansion chamber, and end wave eliminator with the representative blast overpressure by the system. (**B**) Schematic diagram illustrating the configuration of the blast simulator with labeled driver and test sections, membrane position, propagation of the shock wave, ferret orientation during blast exposure, and the end wave eliminator designed to prevent reflection of rarefaction waves back toward the subject.

Primary Blast exposure: Blast exposure procedures followed a previously established protocol using an advanced blast simulator (ABS) (Figure 1A) [16]. Ferrets were anesthetized with 4% isoflurane for 8–10 min and positioned prone, facing the incoming shockwave (Figure 1B). Each animal received two tightly coupled blast exposures at 19 psi, with a 2 min interval between exposures. At either 24 h or 1 month following blast exposure, the animals were humanely euthanized under deep isoflurane anesthesia, and cervical spinal cord segments (C1–C3) were collected. Spinal cords were bisected vertically; half was placed in RNAprotect (Qiagen, Germantown, MD, USA, Cat# 76104) for molecular studies, while the remainder was reserved for Western blot analysis to assess the pathological changes occurring in the spinal cord post-blast [10]. All tissue samples were rapidly frozen and stored at −80 °C until required for further examination.

Gait assessment using the CatWalk XT system: The Catwalk XT system (Noldus Inc., Wageningen, The Netherlands) was utilized to analyze gait changes in ferrets. This automated platform provides precise quantitative evaluation of locomotor function and has been extensively validated for use in spinal cord injury models [20,21,22]. We used the CatWalk system software version XT 9.0 (Noldus Inc., The Netherlands) for analyzing the changes in gait after blast exposure. The Catwalk XT system employs Illuminated Footprint™ technology to record pawprints using a high-speed video camera located beneath the walkway. Offering more than 50 gait and motor function metrics, the catwalk XT’s Illuminated Footprint™ technology also aids in identifying pressure intensity variations between the animal’s paws while walking. In our study, all testing consisted of a minimum of three runs. Although the CatWalk XT has an automatic footprint classification function, the ferret body morphology and toe spread renders it quite ineffective. Hence, the paw print data were manually corrected of errors as required. The data was exported, pooled and the mean of three (or more trials) was calculated using Microsoft Excel for each parameter analyzed.

RNA extraction and quantitative RT-PCR: Total RNA was isolated from tissue samples using the RNeasy Lipid Tissue Mini Kit (Qiagen, Germantown, MD, USA, Cat# 74104) following the manufacturer’s instructions. The extracted RNA was then reverse transcribed into complementary DNA (cDNA) using the RT^2^ First Strand Kit (Qiagen, Germantown, MD, USA, Cat# 330404). A panel of genes including Toll-like receptors (*TLR1*-*TLR10*), cyclooxygenases (*COX-1* and *COX-2*), neurofilament heavy (*NFH*) and light (*NFL*) chains, and key pro-inflammatory cytokines including interleukin-1 beta (*IL-1β*), interleukin-6 (*IL-6*), and tumor necrosis factor alpha (*TNF-α*) were analyzed. Primers were either proprietary assays obtained from Qiagen, USA, or custom-synthesized by Integrated DNA Technologies (IDT), Castle Rock, CO, USA, and Eurofins Scientific, San Diego, CA, USA. Primer sequences used for amplification are provided in Supplementary Table S1. Quantitative real-time polymerase chain reaction (qRT-PCR) was performed using the RT^2^ SYBR Green qPCR Mastermix on an Applied QuantStudio 6 Flex system (Life Technologies, Carlsbad, CA, USA). Each sample was processed in triplicate. Gene expression levels were normalized against β-actin as the endogenous control using the 2^−ΔΔCt^ method. Data are presented as fold changes relative to the sham control group.

Western blotting: Cervical spinal cord tissues were homogenized in T-PER tissue protein extraction reagent buffer supplemented with a protease and phosphatase inhibitor (PPI) cocktail (Millipore Sigma, Burlington, MA, USA, Cat# PPC1010) to preserve protein integrity during extraction. Homogenized samples were centrifuged at 14,000 rpm for 15 min, after which the supernatants were collected. Protein concentration was then measured using the standard bicinchoninic acid (BCA) assay kit (Thermo Fisher Scientific, Waltham, MA, USA, Cat# 23225) according to the manufacturer’s guidelines. Equal amounts of total protein extracts were used for Western blotting using NuPAGE 4–12% Bis-Tris gel (Invitrogen, Carlsbad, CA, USA) and iBlot 2 gel transfer system (Invitrogen, USA). After blocking the membrane with 10% nonfat milk, membranes were incubated with the appropriate primary antibodies followed by horseradish peroxidase (HRP)-conjugated secondary antibodies. Detection was performed using the Pierce^TM^ ECL Western blotting substrate (Thermo Scientific, Waltham, MA, USA, Cat# 32106). The full list of antibodies used is detailed in Supplementary Table S2.

Image Acquisition and Statistical Analysis: Western blot images were acquired using GE Amersham Imager 600 (GE Healthcare, IL, USA), and quantified with Image J software (Version 2.0.0) (National Institutes of Health (NIH), Bethesda, MD, USA). Band intensities were delineated manually with a freehand selection tool to capture each protein band’s area, and densitometric values were normalized to the β-actin loading control for each sample. Statistical comparisons between blast-exposed and sham groups were conducted using unpaired Student’s *t*-tests in GraphPad Prism 8 software (version 8.4.0, GraphPad Software, Boston, MA, USA). Data are presented as mean ± SEM and statistical significance is indicated by asterisks: * *p* < 0.05, ** *p* < 0.01, *** *p* < 0.001.

## 3. Results

### 3.1. Blast Exposure Affected Gait in Ferrets

**Figure 2:**
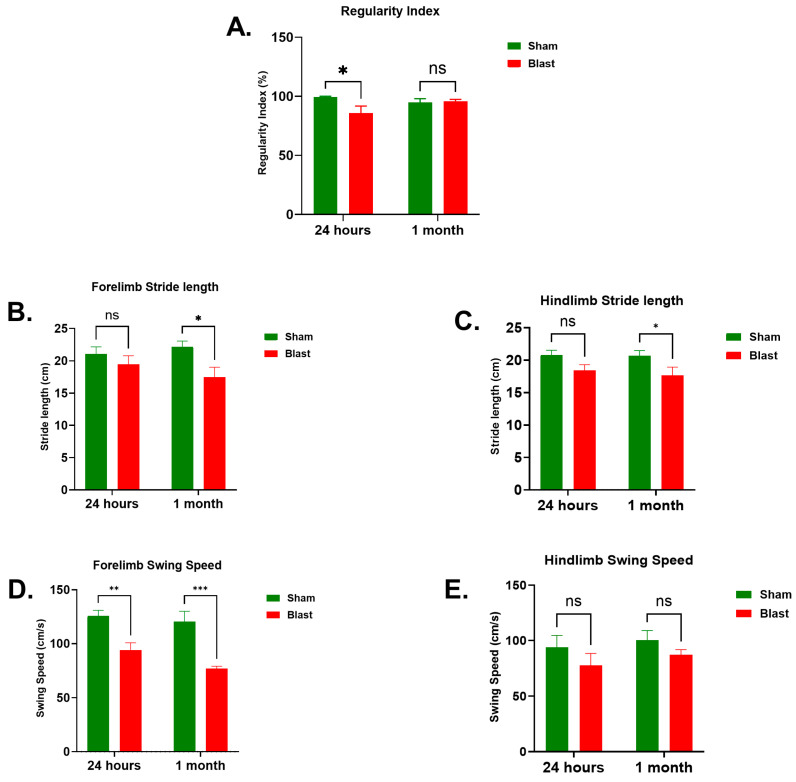
Analysis of gait performance in ferrets at 24 h and 1 month post-blast exposure. Data are represented as mean ± SEM. Regularity index (**A**), stride length in forelimbs and hindlimbs (**B**,**C**), and swing speed in the forelimbs and hindlimbs (**D**,**E**) of the sham ferrets were compared with that of blast exposed ferrets (* *p* < 0.05, ** *p* < 0.01, *** *p* < 0.001, ns—not significant). *n* = 6 to 8 per group/time point.

The CatWalk XT system offers a key advantage in its ability to measure a variety of individual paw and footprint parameters for gait and locomotion analysis. Among these, the regularity index (RI), quantifies interlimb coordination by assessing the proportion of normal step sequence patterns during continuous walking [23]. An RI value of 100% indicates a perfectly coordinated run with exclusive use of normal step sequences. After blast exposure, a statistically significant decrease in the RI, which reflects interlimb coordination during uninterrupted walking, was observed at 24 h compared to the sham controls (*p* = 0.014, Figure 2A). This effect was not maintained at 1 month post-exposure, where RI values did not differ significantly (*p* > 0.05, Figure 2A). Stride length (SL), representing the distance between successive placements of the same paw and indicative of trunk stability and gross muscle strength [24], did not show significant changes in the forelimbs at 24 h post blast exposure (*p* > 0.05, Figure 2B). However, we have noted a statistically significant decrease (*p* = 0.013, Figure 2B) in SL in the forelimbs at 1 month post-blast exposure in comparison to the sham group. In the hindlimbs, we did not see any significant change in SL at 24 h following blast exposure (*p* > 0.05, Figure 2C). However, a statistically significant decrease (*p* = 0.031, Figure 2C) in SL was observed in the hindlimbs at 1 month post-blast exposure in comparison to the sham group. The swing speed (SS) parameter in the CatWalk XT evaluates the velocity of the paw during the swing phase of gait, providing information into the locomotor function and muscle tone of individual limbs. At 24 h post-blast exposure, forelimb SS further decreased relative to sham controls (*p* = 0.003, Figure 2D). This reduction became more pronounced at 1 month post-blast, with forelimb SS further decreasing relative to sham animals (*p* < 0.001, Figure 2D). Notably, hindlimb SS did not exhibit statistically significant differences at either 24 h or 1 month post-blast compared to sham controls (*p* > 0.05, Figure 2E).

### 3.2. Neuroinflammatory Responses in the Cervical Spinal Cord Following Blast Exposure

To evaluate the neuroinflammatory damage post-blast exposure, cervical spinal cord tissue was collected at 24 h or 1 month post-injury. Gene expression profiles of multiple *TLR*s, *COX*, and cytokines were assessed using qRT-PCR. *TLR*s are key mediators of the inflammatory response, activated by diverse exogenous and endogenous ligands, which initiate downstream signaling pathways regulating inflammation and immune gene expression [6]. Activation of TLRs by various exogenous and endogenous ligands leads to the subsequent activation of downstream kinases and cellular signaling pathways, which regulate the expression of genes triggering inflammation and immunity [6]. Hence, we investigated if blast exposure modulates the gene expression of various *TLR*s, *COX*, and cytokine expression in the ferret cervical spinal cord. We examined whether blast injury altered the expression of these inflammatory regulators in the ferret cervical spinal cord.

**Figure 3:**
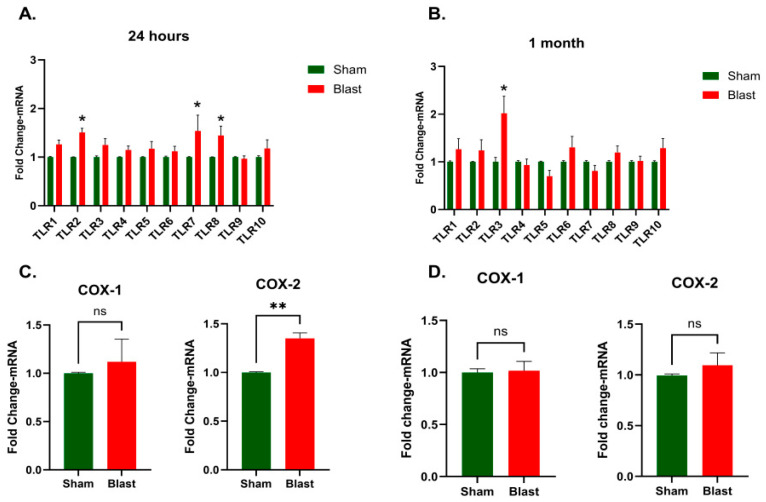
Changes in Toll-like Receptors (*TLRs*) and cyclooxygenase (*COX-2*) gene expression in the ferret cervical spinal cord following blast exposure. mRNA levels of *TLR1-10* (**A**,**B**), COX-1 and COX-2 (**C**,**D**), with results expressed as fold changes relative to the sham (mean ± SEM). Sample size (*n*): 4–8 animals/group/time point. Statistical significance was evaluated by the *t*-test, with asterisks indicating significant differences (** *p* < 0.01; * *p* < 0.05) and ns indicating non-significant.

Results from qRT-PCR analysis demonstrated an increase in several *TLR* transcripts at 24 h post-injury, with *TLR2*, *TLR7*, and *TLR8* showing significant upregulation (*p* < 0.05, Figure 3A). By 1 month, only *TLR3* remained significantly elevated (*p* < 0.05, Figure 3B). Subsequent analyses focused on the expression of *COX-1* and *COX-2* genes to further characterize post-blast inflammatory changes. We have observed a significant upregulation of *COX-2* gene at 24 h (*p* = 0.0012, Figure 3C) and return to near-normal levels at 1 month post-blast injury (*p* = 0.9372, Figure 3D). No statistically significant changes in the *COX-1* gene were noted at either time point (Figure 3C,D).

**Figure 4:**
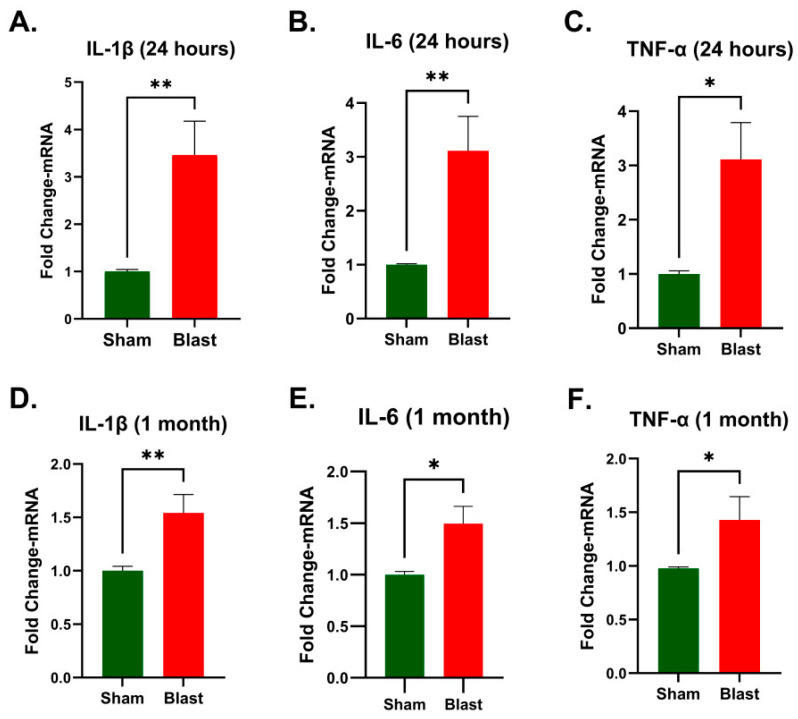
Changes in cytokine mRNA expression in the ferret cervical spinal cord following blast exposure. mRNA expression of *IL-1β* (**A**,**D**), *IL-6* (**B**,**E**), and TNF-α (**C**,**F**). Gene expressions are presented as fold change relative to the sham (mean ± SEM). Sample size (*n*): 5–7 animals/group/time point. Statistical significance was assessed by the *t*-test, with * *p* < 0.05 and ** *p* < 0.01 indicating significant differences compared to the sham control.

Since *TLR* activation drives cytokine gene expression linked to inflammation, the study also assessed the mRNA expression of pro-inflammatory cytokines *IL-1β*, *IL-6*, and *TNF-α*. *IL-6* plays a dual role following spinal cord injury, contributing to both tissue damage and repair, with elevated levels correlating to worse neurological outcomes while moderate expression may promote recovery [25,26,27]. Our data revealed a significant elevation of IL-6 transcripts at both 24 h (*p* = 0.0025, Figure 4B) and 1 month (*p* = 0.0317, Figure 4E) post-blast. Likewise, *IL-1β* and *TNF-α*, major inflammatory cytokines implicated in microglial activation, blood–brain barrier disruption, and neuronal damage [28,29], were significantly upregulated at 24 h [IL-1β, *p* = 0.0025 (Figure 4A); TNF-α, *p* = 0.0177 (Figure 4C)] and remained elevated at 1 month post-blast [IL-1β, *p* = 0.0051 (Figure 4D); TNF-α, *p* = 0.0411, (Figure 4F)]. These findings demonstrate the sustained activation of pro-inflammatory cytokines in the cervical spinal cord during both acute and sub-acute phases post-blast injury.

### 3.3. Neurofilament Alterations Following Acute and Sub-Acute Blast Exposure

**Figure 5:**
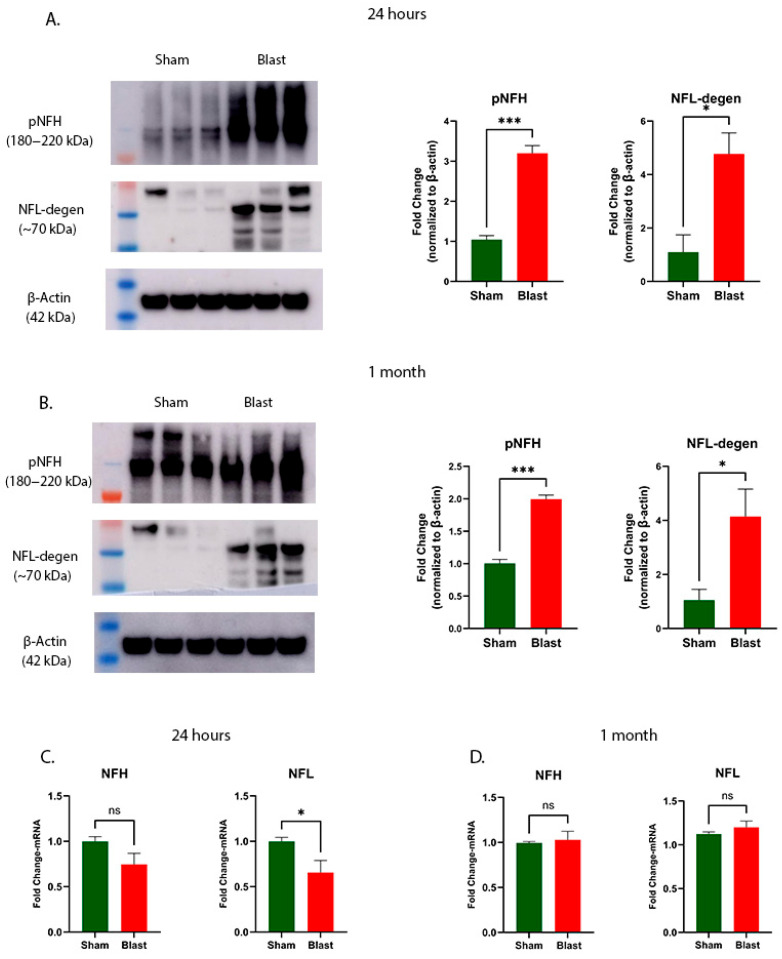
Effects of blast exposure on neurofilaments in the ferret cervical spinal cord. Representative Western blot for pNFH and NFL-degen relative levels are shown, with protein levels normalized to β-actin and presented as fold change relative to the sham controls (mean ± SEM) (**A**,**B**). mRNA expression of *NFH* and *NFL* (**C**,**D**). Sample size (*n*): 6 animals/group/time point. Statistical significance was determined using Student’s *t*-test; *** *p* < 0.001 and * *p* < 0.05 denote significant differences versus sham, while ns indicates no significant difference.

Neurofilaments are neuron-specific cytoskeletal proteins playing important roles in growth, structure and stability of axons [30]. Neurofilament proteins exist in three main subtypes, distinguished by their molecular weights: the light chain (NFL) at 68 kDa, the medium chain (NFM) at 150 kDa, and the heavy chain (NFH), which is about 200 kDa. Phosphorylated NFH (pNFH) has been demonstrated to indicate damaged and demyelinated axons, and various lines of evidence suggest that blast exposure can lead to the significant damage of neurofilaments (an essential component of the neuronal cytoskeleton) in the brain [31]. Hence, we investigated the pNFH protein expression in the cervical spinal cord following blast exposure by Western blotting. A statistically significant elevation in pNFH protein levels was detected in the spinal cord at 24 h (*p* = 0.0006, Figure 5A) and persisted at 1 month (*p* = 0.0004, Figure 5B) post-blast exposure. The gene expression analysis of NFH using qRT-PCR did not show statistically significant changes in the spinal cord at 24 h (*p* = 0.0823, Figure 5C) or 1 month (*p* = 0.9372, Figure 5D) after blast exposure.

Similarly to pNFH, blast injury causes damage to neuronal axons in the brain and leads to the release of NFL into the bloodstream [32,33]. NFL levels in the spinal cord have been recognized as a biomarker indicative of axonal injury following blast exposure [10]. We utilized the NFL DegenoTag^TM^ (NFL-degen) antibody [34], which specifically identifies NFL present in the neurons undergoing degeneration to evaluate potential neurodegeneration occurring in the cervical spinal cord following blast exposure. We noticed a statistically significant rise in immunoreactivity for the NFL-degen antibody, which marks degenerating neurofilament light chains, suggesting accelerated neurodegeneration in the spinal cord. These increases were evident at both 24 h (*p* = 0.0229, Figure 5A) and 1 month (*p* = 0.0477, Figure 5B) after blast exposure. The gene expression analysis of NFL using qRT-PCR showed statistically significant decrease in the spinal cord at 24 h (*p* = 0.0262, Figure 5C), but not at 1 month (*p* = 0.2273, Figure 3D) after blast exposure.

### 3.4. Blast Exposure Causes Tau Hyperphosphorylation at Acute and Sub-Acute Time Points

**Figure 6:**
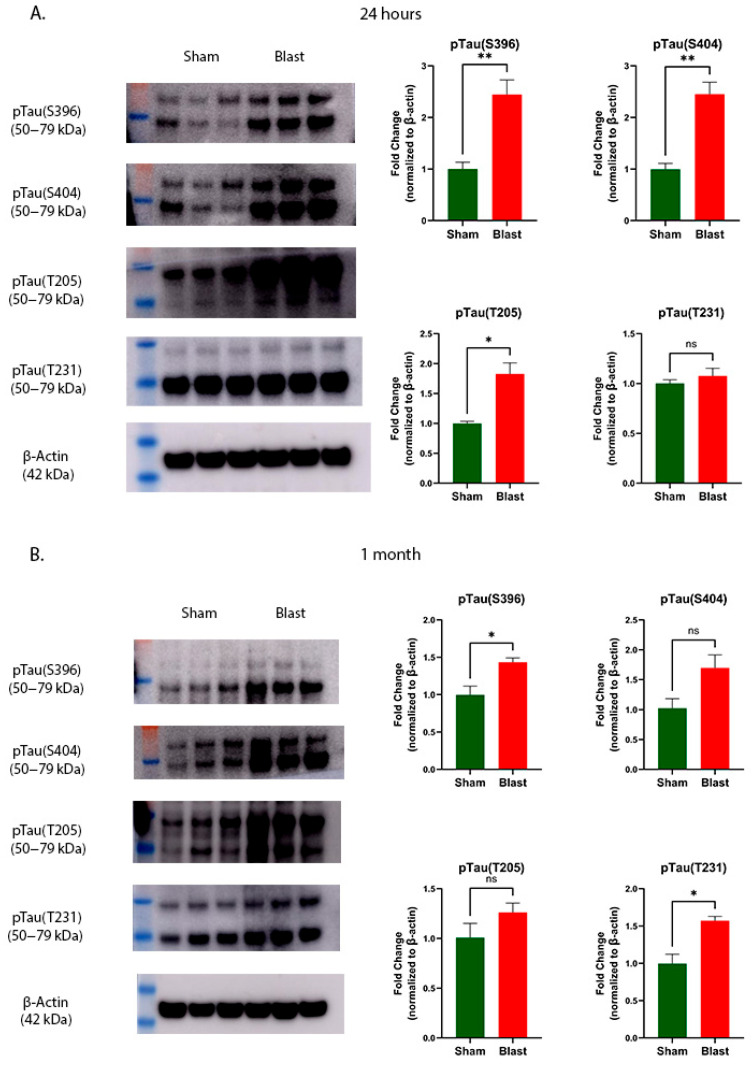
Effects of blast exposure on Tau protein phosphorylation in ferret cervical spinal cord. Representative Western blot images show pTau(S396), pTau(S404), pTau(T205), and pTau(T231) levels normalized to β-actin. Quantitative analysis is presented as fold change relative to sham controls at 24 h (**A**) and 1 month (**B**) (mean ± SEM). Sample size (*n*): 6 animals/group/time point. Statistical significance was evaluated using Student’s *t*-test, with asterisks indicating significant differences (** *p* < 0.01; * *p* < 0.05). ns indicates no significant difference.

Abnormal hyperphosphorylation of the Tau protein reduces its microtubule-binding capacity and disrupts microtubule stability, ultimately leading to neuronal damage and cell death. A hallmark of neurodegeneration following spinal cord injuries not related to blasts is the hyperphosphorylation of Tau at various serine and threonine residues [35,36]. Hence, we measured the phosphorylation status of the Tau protein at various specific amino acids (Ser396, Ser404, Thr231, and Thr205). As shown in Figure 6A, blast exposure significantly increased the phosphorylation of Tau protein at Ser396 (*p* = 0.0098) and Ser404 (*p* = 0.0049) in the cervical spinal cord compared to the respective sham groups at 24 h. In addition to serine phosphorylation, threonine residues showed hyperphosphorylation at Thr205 (*p* = 0.0122), but not at Thr231 (*p* = 0.4367). At 1 month post-blast exposure, the hyperphosphorylation of Tau at Ser396 (*p* = 0.0281) and Thr231 (*p* = 0.0148), but not at Ser404 (*p* = 0.0685) and Thr205 (*p* = 0.2113) was observed (Figure 6B).

### 3.5. Blast Exposure Alters GFAP and Iba-1 Protein Expression in the Cervical Spinal Cord

**Figure 7:**
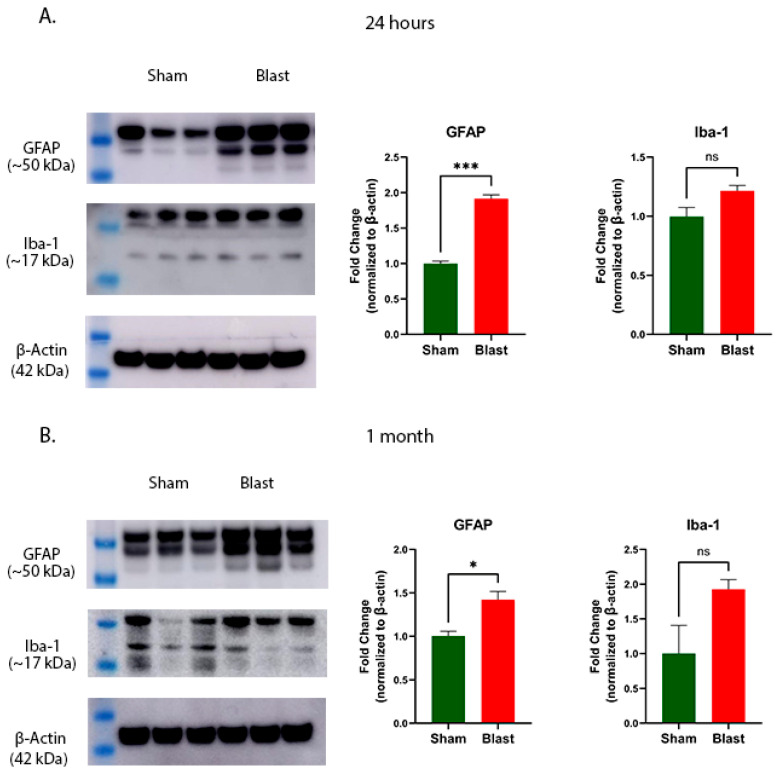
Effects of blast exposure on GFAP and Iba-1 protein levels in the ferret cervical spinal cord. Representative Western blot images showing GFAP and Iba-1 protein levels, normalized to β-actin, are presented as fold changes relative to the sham group at 24 h (**A**) and 1 month (**B**) (mean ± SEM). Sample size (*n*): 6 animals/group/time point. Statistical comparisons were made using Student’s *t*-test; significant differences relative to the sham are indicated by *** *p* < 0.001 and * *p* < 0.05, while ns denotes no significant difference.

Military blast exposure has been shown to trigger prominent neuroinflammation, characterized by increased reactive astrogliosis, microgliosis, and elevated pro-inflammatory cytokines in the brain and spinal cord during early post-injury periods [37,38,39]. In this study, we assessed both the immediate and longer-term effects of the blast on glial activation markers in the ferret cervical spinal cord. Our findings indicate a significant elevation in GFAP, an indicator of astrogliosis, in blast-exposed animals compared to sham controls at 24 h (*p* = 0.0001, Figure 7A) and at 1 month post-injury (*p* = 0.0182, Figure 7B). Although Iba-1, a marker for microglial activation, showed a trend towards increased expression, its levels did not reach statistical significance at either 24 h (*p* = 0.0722, Figure 7A) or 1 month (*p* = 0.0966, Figure 7B) following blast exposure.

## 4. Discussion

This study represents the first preclinical investigation into the neuropathological effects of blast injury on the spinal cord using a gyrencephalic animal model, which more closely mirrors the human central nervous system compared to traditional rodent models. By examining both acute and sub-acute changes in the cervical spinal cord following blast exposure, this study emphasizes the involvement of multiple *TLR*s and COX-2 in promoting the expression of pro-inflammatory cytokines and markers of axonal injury. Key findings supporting these conclusions include the following: (1) alterations in several gait parameters after blast exposure; (2) increased gene expression of *TLR2*, *TLR7*, *TLR8*, along with elevated levels of *IL-1β*, *IL-6*, and *TNF-α* in spinal tissue; (3) elevated pNFH protein and enhanced immunoreactivity to neurofilament light chain degeneration markers; (4) hyperphosphorylation of Tau protein at multiple serine and threonine sites; and (5) increased GFAP expression indicating astrogliosis. These results provide valuable insights into potential mechanisms driving axonal degeneration in blast-induced traumatic spinal cord injury. Given that blast-related spinal cord injuries frequently impair gait through effects such as reduced walking speed, altered cadence, shorter steps, exaggerated knee flexion during initial contact, increased trunk sway, and changes in pelvic movement, understanding molecular and cellular changes in the cervical spinal cord, which houses complex neural circuits frequently affected in human spinal cord injury, is of critical importance. The CatWalk XT system is an automated tool designed for the comprehensive analysis of spatial and temporal gait parameters related to sensorimotor performance and interlimb coordination, both of which can be affected by spinal cord injury. A notable feature of this system is its capability to assess multiple parameters for each individual paw or footprint, making it a highly useful method for functional evaluation of spinal injuries in animal models. Beyond blast injuries, the CatWalk XT has also been widely applied to detect gait disturbances in rodent models with neurodegenerative diseases and other brain or spinal cord injuries [21,40,41,42]. The regularity index (RI) indicates the interlimb coordination of animals during uninterrupted locomotion. In our study, we observed a reduction in RI at 24 h post-blast exposure, reaching near-normal levels at 1 month post-blast. In a cervical clip contusion/compression model of SCI, a significant decrease in the RI levels at an acute time point was noted with slow recovery after a few weeks. Similarly, other studies have also reported decrease in the RI levels following SCI [43]. The decrease in the RI in our blast model is smaller compared to the contusion-induced SCI models due to the lower severity of injury to the spinal cord after the moderate-level blast exposure. The RI is one of the most popular gait parameters used for functional testing after SCI due to its high sensitivity.

Stride length (SL) refers to the distance between two successive placements of the same paw during locomotion, providing an indication of trunk stability and overall muscle strength. Our current results revealed that SLs in the forelimbs and hindlimbs were significantly reduced at 1 month post-blast indicating disturbances in the locomotor functions after the SCI. Furthermore, recent research indicates that SL along with a base of support parameters indicate specified use other than locomotion assessment which possibly explains the subtle changes in SL following blast exposure [44,45,46,47].

The swing speed (SS) is a gait measure based on a single limb that indicates the paw’s speed during a swing, offering insights into absolute velocity and acting as a crucial sign of movement and coordination problems. A notable decrease in swing speed (SS) of the forelimbs was observed at both evaluated time points. However, no significant alterations in hindlimb SS were detected. These findings suggest that the forelimb motor circuits within the cervical spinal cord may be more extensively affected by blast exposure, potentially accounting for the differences observed in spontaneous recovery between the forelimbs and hindlimbs at one month post-injury. Notably, the gait parameters analyzed in this study exhibited significant reductions in blast-exposed ferrets compared to shams, regardless of the specific limbs measured. Nevertheless, a deeper understanding of these gait parameters along with other functional tests needs to be performed to validate the extent of blast-related injury to the spinal cord.

Blast exposure has been shown to upregulate multiple *TLRs* in different brain regions of ferrets, indicating an activated inflammatory response following blast injury [16]. Increases in mRNA levels of *TLR*s (*TLR1*, *2*, *4*, *5*, and *7*) and their downstream signaling molecules have been observed in mouse models of spinal contusion injury [8]. Another study using a murine contusion model reported the upregulation of various TLRs at different time points post-injury [48]. Specifically, *TLR1* and *TLR2* expression increased at 6 hours post-injury, while *TLR1*, *TLR2*, *TLR4*, *TLR6*, and *TLR7* showed elevated levels at 48 hours compared to sham controls [48]. No previous studies in any animal models of blast exposure determined the differential expression of *TLR*s in the spinal cord. Neuroinflammatory changes, specifically changes in several interleukins and TNF-α, were noted in the brain following single and repeated blast exposures [49]. We have observed an increase in the mRNA expression of *TLR2*, *TLR7* and *TLR8*, suggesting stimulation of the *TLR*-dependent neuroinflammatory pathway in the spinal cord at 24 h post-blast injury, prompting consideration that therapeutic agents which can inhibit multiple *TLR*s may be efficacious as potential countermeasures. Since the upregulation of *TLR3* was observed in the spinal cord at 1 month, but not at 24 h post-blast, *TLR3* might play roles in the secondary effects of bSCI, and inhibitors specific to *TLR3* may be useful in preventing secondary effects.

In this study, there were no significant changes observed in *COX-1* mRNA levels at either 24 h or 1 month after bSCI. On the other hand, *COX-2* expression showed a significant increase at 24 h post-injury, suggesting that *COX-2* selective inhibitors, such as meloxicam, might offer therapeutic benefits in reducing spinal cord damage following blast exposure. This pattern of *COX* expression aligns with findings from a rat organotypic hippocampal model of repeated bTBI, where *COX-2* levels rose at 48 h after injury while *COX-1* remained unchanged [50]. Also, elevated *COX-2* expression has been reported in non-bTBI models [51,52,53]. While *COX-1* is constitutively expressed under normal conditions and participates in physiological functions within the brain, *COX-2* is typically induced during pathological states. Thus, the specific upregulation of *COX-2* following bSCI implies its predominant role in driving neurological impairment. Studies involving brain injury models without TBI, such as excitotoxin or amyloid-β peptide injections, similarly suggest that pathological *COX-2* induction may contribute to neurological deficits [54,55].

Axonal white matter injury is a critical determinant of outcomes following mild and severe TBI [56]. However, studies examining blast-induced axonal damage specifically in the spinal cord remain limited. NFL, NFM, and NFH form the structural framework of axons and dendrites, playing vital roles in their branching and growth [57]. In the context of TBI, neurofilament levels have emerged as valuable diagnostic and prognostic biomarkers. pNFH, a large protein with a molecular weight ranging from 190 to 210 kDa depending on phosphorylation status, is released from injured neurons into extracellular spaces, subsequently entering CSF and blood circulation [58,59]. Our findings revealed elevated pNFH protein expression in the cervical spinal cord at both 24 h and 1 month post-blast. Previous investigations also showed that blast exposure induces axonal degeneration in the rat brain acutely and chronically, leading to increased pNFH levels in CSF [31]. Clinically, raised blood levels of pNFH correlate with axonal damage severity in TBI patients [58]. Similarly, rodent blast injury models demonstrate increased pNFH immunoreactivity in cortical and hippocampal regions from 18 h up to 7 days post-injury, suggesting disrupted axonal transport [60,61]. In parallel, the disruption of axonal transport manifests as abnormalities in NFL, visible as swollen axons with altered morphology. NFL immunohistochemical staining has been employed to assess axonal injury within the cervical spinal cord after blast exposure [10]. Swollen NFL-positive axons exhibiting vacuolations and retraction bulbs have been documented during acute post-blast stages (6 and 24 h) [10]. Consistent with this, our study observed increased immunoreactivity for the NFL-degen antibody, which specifically labels degenerating NFL proteins, indicating that blast exposure promotes neurodegeneration in the cervical spinal cord. Supporting this, a mouse model of experimental autoimmune encephalomyelitis demonstrated that elevated serum NFL correlated with increased NFL-degen positive axons in spinal lesions [62].

Tau protein has been identified as a marker of axonal injury in TBI studies [63,64]. TBI can cause widespread axonal damage and compromise the blood–brain barrier, leading to the disruption of Tau’s normal association with tubulin [65]. This disturbance results in hyperphosphorylated Tau accumulating as oligomers within neurons. Such abnormal Tau phosphorylation and deposition are hallmark features of chronic traumatic encephalopathy (CTE) and neurodegenerative diseases like Alzheimer’s. Notably, hyperphosphorylated Tau aggregates, known as neurofibrillary tangles, have been observed in the brains of military veterans who experienced blast-related TBI [66,67]. Various blast injury animal models have also consistently demonstrated elevated Tau phosphorylation in brain regions from 24 h to several months following injury [66,68,69,70,71]. Although Tau hyperphosphorylation in the spinal cord has been reported after traumatic injuries unrelated to a blast, data on its expression in the spinal cord post-blast exposure is lacking. In this study, increased phosphorylation of Tau at serine 396, serine 404, and threonine 205 was detected at 24 h post-blast and persisted, albeit to a lesser extent, at one month. The sustained elevation of phosphorylated Tau at one month suggests that blast injury triggers early and lasting Tau-associated neurodegeneration in the cervical spinal cord. Supporting literature reports increased the phosphorylation of Tau at multiple residues in mouse brain regions after blast exposure, with changes lasting at least 30 days [69]. Other studies also note transient elevations of phosphorylated Tau in the brain following a blast, which gradually diminish over weeks to months post-injury. These results reinforce the idea that blast exposure may initiate pathological Tau aggregation, contributing to neurodegenerative processes in the spinal cord similar to those observed in the brain. In non-blast traumatic spinal cord injuries, hyperphosphorylated Tau accumulates and spreads throughout the spinal cord and cerebrospinal fluid, reflecting widespread Tau pathology involving the brain [36]. Elevated Tau levels in serum and CSF have likewise been associated with injury severity and functional outcomes in various spinal cord injury models [35,72]. Furthermore, therapeutic inhibition of Tau has been proposed as a strategy to enhance recovery following spinal cord injury [73].

We evaluated glial activation in the cervical spinal cord after blast exposure by measuring protein levels of GFAP, an astrocyte marker, and Iba1, a marker for microglia, at acute and sub-acute time points. GFAP is widely recognized in clinical settings as a biomarker for TBI [74], where its increased expression reflects astrocyte activation and proliferation, contributing to glial scar. Our study found elevated GFAP expression at 24 h post-blast injury, which remained significantly higher at 1 month, consistent with prior findings showing astrogliosis in the brains of individuals with cortical contusion injuries [75]. Iba1 protein levels showed only a slight, non-significant increase at both time points, possibly due to the relatively low blast overpressure used in our model. Previous research has demonstrated a robust activation of astrocytes and microglia in the brain following blast exposure [76,77], and similar glial responses have been observed in spinal cord contusion injuries [78,79]. Earlier studies in rodent models with smooth (lissencephalic) brains reported spinal cord neuroinflammation following a blast [39] but focused mainly on acute effects, noting an increased expression of both GFAP and Iba1 at 72 h, an interval not included in our analysis. A key limitation of that prior research was the use of artificially focused blast waves targeting the spinal cord, differing substantially from the whole-body blast exposures typically experienced by humans [80]. Although the ABS system used in this study is specifically designed to eliminate artefactual blast wind and the ferrets were positioned in a fixed, longitudinal orientation to ensure that the blast waves impact the head directly, mild head/cranial movement as a confounding factor for the cervical spinal cord injury cannot be ruled out. We did not investigate the role of kinases and phosphatases in mediating Tau hyperphosphorylation. Further studies investigating the phosphorylation of Tau and related enzymatic activities at acute and chronic time points following single and multiple blast exposures will be needed to determine if the Tau hyperphosphorylation observed in our model is a reversible phenomenon (or not) and which molecular mechanisms control it. In addition, more objective methods to assess locomotor changes using different functional tests along with histological assessment in the form of white and gray matter changes, inflammatory infiltration, and immunohistochemistry are useful additions. Although such assessments could provide additional mechanistic insights, the current approach still provides a robust and quantitative evaluation of gait deficits. Even though the time points evaluated in this study provide us with crucial information on acute and sub-acute functional changes, long-term assessments would be more valuable as blast-related neurodegeneration can progress over several months or even years. Further consideration needs to be taken into account for the genetic and environmental factors such as gender, age, and pre-blast conditions that could contribute to further detrimental effects in addition to those triggered by the initial blast events.

## 5. Conclusions

In summary, the results obtained from this study suggested that blast overpressure waves can induce changes in gait performance, neuroinflammation, and axonal injury in the cervical spinal cord of a gyrencephalic animal at acute and sub-acute time points post-blast. The acute increase in the expression of multiple *TLR*s supports the notion that therapeutic interventions targeting multiple *TLR*s will be more effective than focusing on a single *TLR* in protecting the spinal cord against blast exposure. The results from this study also support the acute use of *COX-2* inhibitors in preventing the primary injury events, and inhibitors specific to *TLR3* might prevent the secondary injury mechanisms in the spinal cord post-blast. Changes in the markers of axonal injury and resulting neuronal degeneration observed in the cervical spinal cord suggested similar pathological mechanisms reported earlier in the brain post-blast.

## Data Availability

Material has been reviewed by the Walter Reed Army Institute of Research. There is no objection to its presentation and/or publication. The opinions or assertions contained herein are the private views of the author, and are not to be construed as official, or as reflecting the true views of the Department of the Army or the Department of Defense. Research was conducted under an IACUC-approved animal use protocol in an AAALAC International-accredited facility with Public Health Services Animal Welfare Assurance, and in compliance with the Animal Welfare Act and other federal statutes and regulations relating to laboratory animals.

## Supplementary Materials

The following supporting information can be downloaded at https://www.mdpi.com/article/10.3390/brainsci15101050/s1, Table S1: Primer information for quantitative RT-PCR assays; Table S2: List of antibodies.
