# Supplementary material for: Blast Overpressure-Induced Neuroinflammation and Axonal Injury in the Spinal Cord of Ferrets
Source: Brain Sci. 2025 Sep 26;15(10):1050. doi: 10.3390/brainsci15101050 (PMC12564047; doi:10.3390/brainsci15101050)
Supplement: Supplementary file 1 [file brainsci-15-01050-s001.zip › brainsci-3818274-supplementary.pdf]

Supplementary Table S1. Primer information for quantitative RT-PCR assays.

Gene	Source	Product/Accession Number	Forward Primer (5'→3')	Reverse Primer (5'→3')
β-Actin	Qiagen	XM_004775013.2_1	-	-
TLR1	Qiagen	XM_013062225.2_1	-	-
TLR2	Qiagen	XM_004771998.2_1	-	-
TLR3	Qiagen	XM_013059014.1_1	-	-
TLR4	Qiagen	XM_004774068.2_1	-	-
TLR5	Qiagen	XM_045076697.1_1	-	-
TLR6	Qiagen	XM_045076532.1_1	-	-
TLR7	Qiagen	XM_004758838.3_1	-	-
TLR8	Qiagen	XM_045089302.1_1	-	-
TLR9	IDT	-	ACTCCGACTTTGTCCACCTG	GGTCATGTGACAGGGGAAGT
TLR10	IDT	-	CTGTTTTCAGGTGCTTGCCC	AATGGCACCACTCACTCTGG
COX-1	IDT	-	CATCCATCTACTCCCAGAGTCATGAG	GAGGGCTGGGGATAAGGTTGGACCGC
COX-2	IDT	-	GATTGACAGCCCACCAACTT	CGGGATGAACTCTCTCCTCA
NFH	Eurofins	-	GAGGAGTGGTTCCGAGTGAGAC	ATGACGGTCCTCCAGCTCA
NFL	Eurofins	-	ATCAGTGCTATGCAGGACACAAT	GAGTCGGGTCTCCTCACCTTC
IL-1β	Eurofins	-	TTTCTAAAGCAGCCATGGCA	CTTCTACTCCCTTTCCATCAG
IL-6	Eurofins	-	CAAATGTGAAGACAGCAAGGAGGCA	TCTGAAACTCCTGAAGACCGGTAGTG
TNF-α	IDT	-	CCAGATGGCCTCCAACTAATCA	GGCTTGTCACTTGGAGTTCGA

Note: Proprietary Qiagen primers are referenced by catalog numbers, without disclosing primer sequences. For custom primers sourced from IDT and Eurofins, exact forward and reverse sequences (5′ to 3′) are provided.

Supplementary Table S2: List of antibodies

Primary Antibody	Catalog Number	Company	Dilution
**pNFH**	RPCA-NFH	EnCor Biotechnology	1:20000
**NFL-degen**	MCA-6H63	EnCor Biotechnology	1:10000
**pTau (ser396)**	ab109390	Abcam	1:5000
**pTau (ser404)**	20194	Cell Signaling	1:1000
**pTau (Thr205)**	44-738G	Invitrogen	1:5000
**pTau (Thr231)**	701056	Invitrogen	1:5000
**GFAP**	RPCA-GFAP	EnCor Biotechnology	1:10000
**Iba-1**	019-19741	Wako	1:500
**β-Actin**	A3854	Sigma	1:20000
**Secondary Antibody**			
**Goat Anti-Rabbit**	65-6120	Invitrogen	1:2500
**Goat Anti-mouse**	5220-0341	Sera care	1:2500
